# Oropharyngeal dysphagia in dermatomyosites: case report and literature review

**DOI:** 10.1016/S1808-8694(15)30157-9

**Published:** 2015-10-18

**Authors:** Elza Maria Lemos, Patricia Paula Santoro, Raquel Aguiar Tavares, Roberta Ismael Dias Garcia, Cristina Lemos Barbosa Furia

**Affiliations:** a Graduate student - Otorhinolaryngology - HCFMUSP, Head of the Pediatric Dysphagia Ward - HCFMUSP (University of São Paulo Medical School; b PhD in Medicine - FMUSP. Assistant Otorhinolaryngologist - Otolaryngology Ward - HCFMUSP, Head of the Dysphagia Ward - HCFMUSP; c Graduate Student in Otorhinolaryngology - HCFMUSP, Otorhinolaryngologist; d Graduate Student in Otorhinolaryngology - HCFMUSP, Otorhinolaryngologist; e PhD in Oncology - FMUSP, Speech and Hearing Therapist - Speech and Hearing Department - - HCFMUSP

**Keywords:** dermatomyosites, dysphagia, videoendoscopic study, swallowing

## Abstract

We present a rare case of dermatomyosites associated with severe oropharyngeal dysphagia. A 13 year old female patient, being followed at the Rheumatologic Department, was referred to the Otolaryngology Department of the University of São Paulo School of Medicine Hospital. She complained of swallowing problems, especially with solids. Following our dysphagia study protocol, we employed a speech pathologist and otolaryngology evaluation, mainly for clinical history, examination of anatomical structures involved with swallowing events, cranium nerves integrity and videoendoscopic swallowing study. We diagnosed severe oropharyngeal dysphagia, with aspiration of saliva and food of all consistencies. We advised against oral feeding and recommended a diet through a gastric tube. She started with therapy and xerostomia medication, together with the treatment of the base disease. The patient showed a significant improvement, noticed by the clinical evaluation and the control videoendoscopic swallowing study, with the possibility of returning to oral feeding. The authors stress the incidence of oropharyngeal dysphagia in dermatomyosites and suggest the videoendoscopic swallowing study as a good exam for diagnosis and follow-up of these patients.

## INTRODUCTION

It is rare to find oropharyngeal dysphagia in dermatomyositis (DM) in the literature1. We present a case of DM with severe oropharyngeal dysphagia. DM represents a muscular inflammatory disorder, with a characteristic skin rash. It is more frequent in females (3:1). It has two incidence peaks: in childhood and in the fifth decade of life¹.

The fundamental clinical aspect is proximal and symmetrical muscle weakness, involving the pelvis and shoulder blade, anterior neck and trunk flexors. In severe cases, the weakness can be widespread^2^.

Dysphagia is present in about 15% of the patients, and happens because of an involvement of the pharyngeal and upper esophagus striated muscles. When present, it predisposes the patient to aspiration pneumonia - one of the major causes of death^1^.

Diagnosis is based on clinical aspects associated with complementary tests: enzymes, electromyography and biopsy. Numerous evidences claim its classification as autoimmune^2^.

Treatment of choice is prednisone or other nonfluorinated steroids^3^.

Out of the acute phase, physical therapy or occupational therapy help in the recovery of muscle strength.

## CASE PRESENTATION

E.A.P.N., Caucasian, 13 year-old female with a diagnosis of acute DM. Complained of difficulties to swallow solids, sialorrhea, gagging, productive cough, voice change and weight loss for 30 days. Unable to feed orally, she had been receiving parenteral diet through a naso-gastric tube since the disease onset.

Speech assessment showed: reduction in muscle tone and strength of the speech movement muscles, residues remaining in the oral cavity, wet voice after swallowing food of all consistencies, noisy neck auscultation before and after swallowing.

**Figure 1:**
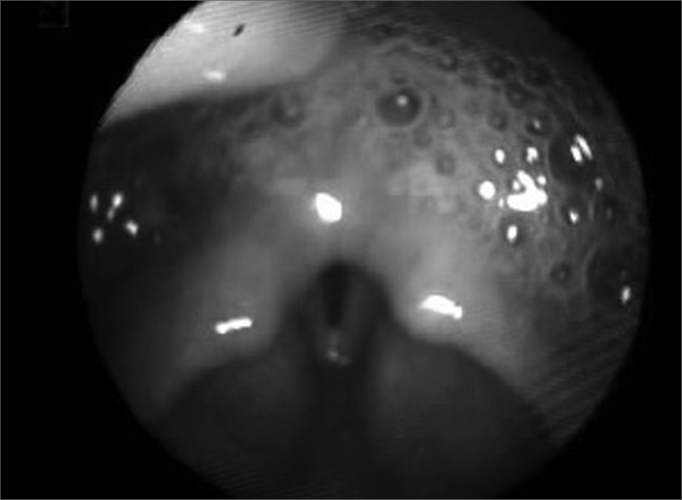
Saliva stasis - Swallowing videoendoscopy exam, showing the presence of saliva stasis in the pyriform recesses, in the retrocricoid area and signs of aspiration.

Otorhinolaryngological evaluation through nasal fibroscopy showed pharyngeal muscle incompetence, abundant saliva stasis in the pyriform recess, with signs of aspiration (Figure 1). We added swallowing videolaryngoscopy,^4^, ^5^ which showed: laryngotracheal penetration and aspiration of all the samples swallowed and cough reflex after aspiration. We then diagnosed severe oropharyngeal dysphagia, and instructed the patient to keep feeding through the naso-gastric tube and start physical therapy with a speech specialist. We also suggested the use of 25mg-day amitriptyline to induce xerostomia and reduce saliva aspiration.

Speech therapy aimed at increasing the strength of oropharyngeal structures and saliva swallowing, and posture maneuvers training after improvement.

After eight weeks, the patient was swallowing better and a new swallowing videolaryngoscopy showed moderate dysphagia. We decided to gradually increase oral feeding, keeping the naso-gastric feeding. After 17 weeks a third swallowing videolaryngoscopy showed that the moderate dysphagia remained.

The patient remained under otorhinolaryngological and speech therapy for six months and was able to oral feed properly, we then removed the naso-gastric tube.

## DISCUSSION

The patient presented symptoms of dysphagia in the beginning, causing important weight loss and malnourishment. In this disorder it is important to perform a guided interview, using the swallowing disorders questionnaire, because these patients do not complain of spontaneous swallowing difficulties^6^.

Literature describes dysphagia improvement after pulse therapy, with symptoms disappearing in 2 to 14 days^3^. In this case, we observed a long standing disorder, and the patient had already received pulse therapy with general improvement. Being followed for six months, the patient was able to control saliva aspiration, however without being able to oral feed exclusively.

Speech therapy brought about benefits as strength gain and swallowing effectiveness. Xerostomia reduced laryngotracheal penetration and aspiration of saliva.

## FINAL REMARKS

It is important to highlight how few papers we have in the literature that discuss dysphagia specifically in this group of patients. It is necessary to pay attention to the interview protocol and the clinical evaluation.

The authors hereby present the work of a multidisciplinary team in the diagnosis and follow up of dysphagia, with sequential evaluations, allowing diet progression in a safe and effective way, reducing the risks of aspiration pneumonia^4^.
